# Efficacy and safety of two artificial saliva-based polymers containing 0.1% pilocarpine for treatment of xerostomia: A randomized clinical pilot trial

**DOI:** 10.4317/jced.58415

**Published:** 2021-10-01

**Authors:** Wilairat Sarideechaigul, Aroonsri Priprem, Sucharat Limsitthichaikoon, Pensri Phothipakdee, Rajda Chaijit, Teekayu P. Jorns, Nopphakhun Lungruammit, Krittiporn Chaiya

**Affiliations:** 1 Department of Oral Biomedical Science, Faculty of Dentistry, Khon Kaen University, Khon Kaen 40002, Thailand; 2 Neuroscience Research and Development Group, Khon Kaen University, Khon Kaen 40002, Thailand; 3 Faculty of Pharmacy, Mahasarakham University, Mahasarakham 44150, Thailand; 4 Melatonin Research Group, Khon Kaen University, Khon Kaen 40002, Thailand; 5 Department of Pharmaceutical Technology, College of Pharmacy, Rangsit University, Pathum Thani 12000, Thailand; 6 Department of Preventive Dentistry, Faculty of Dentistry, Khon Kaen University, Khon Kaen 40002, Thailand; 7 Dental Student, Faculty of Dentistry, Khon Kaen University, Khon Kaen 40002, Thailand

## Abstract

**Background:**

Topical agents are the mainstay in the treatment of xerostomia, a common complaint most frequently associated with salivary dysfunction. This study aimed to compared the efficacy and safety for xerostomia treatment of 2 artificial saliva preparations containing 0.1% pilocarpine, and, either sodium carboxymethylcellulose (SCMC), or, sodium polyacrylate (SPA).

**Material and Methods:**

Thirty-one xerostomia patients were randomly allocated into either a SCMC-treated group (15 patients), or, a SPA-treated group (16 patients). The formulations were taken 0.5 ml, 4 times daily for 6 weeks and double-blinded assessed before and after treatments using Xerostomia Inventory (XI) and Clinical Oral Dryness Score (CODs). Unstimulated and stimulated whole salivary flow rates were measured.

**Results:**

After treatment, the SCMC-treated group had significantly lower CODs and higher unstimulated and stimulated whole salivary flow rates (*p*<0.001, *p*=0.035, and *p*=0.013, respectively), while the SPA-treated group showed significantly lower CODs only (*p*=0.004). In contrast, SCMC-treated and SPA-treated groups at the 6th week after treatments showed non-significant differences in all assessments (*p*>0.05, all). Some adverse events (AEs) were reported, e.g., burning tongue, dizziness and watery eyes, but no severe AEs.

**Conclusions:**

This randomized controlled pilot trial demonstrated superior efficacy of SCMC-formula over a SPA-formula after 6 weeks of xerostomia treatment. These formulations with topical pilocarpine proved safe in clinical use with minimal reported AE.

** Key words:**Xerostomia, artificial saliva, sodium carboxymethylcellulose, sodium polyacrylate, pilocarpine.

## Introduction

Xerostomia is defined as the subjective perception of dry mouth, whereas salivary gland hypofunction (SGH) is the objective reduction of salivary flow and changes in its composition (1). These can affect chewing, swallowing, speaking, denture wearing and general well-being. The symptoms include oral dryness, burning sensations and taste alteration. Consequently, oral mucosa can become dry and atrophy. Patients can present with significant dysgeusia, dysphagia and dysarthria, and an increased risk of developing oral ulcerations, dental caries, periodontal diseases, oral candidiasis, and bacterial sialadenitis (2). The prevalence of xerostomia increases dramatically with age, mainly affecting middle-aged and elderly populations. The prevalence of xerostomia in population-based studies ranged from 10 to 46%, with a lower prevalence for men (9.7–25.8%) than women (10.3–33.3%) (3). The common etiologies of xerostomia use of xerogenic medications (such as antidepressants, antihypertensives, anticholinergic agents, antihistamines, and hypoglycemics), ageing, radiochemotherapy and systemic diseases such as diabetes mellitus, chronic renal failure, scleroderma, lupus erythematosus, and Sjögren’s syndrome. Other factors can be depression, anxiety, or stress (4).

The general goals for xerostomia treatment are to alleviate symptoms, prevent the consequences of salivary dysfunctions, and to treat systemic diseases. Symptomatic xerostomia management depends on the degree of salivary dysfunction. These can be divided into endogenous approaches, including enhancing salivary gland function through additional pharmaceutical compounds such as pilocarpine and exogenous approaches such as oral moisturizing and artificial saliva (5). Several studies have been conducted using artificial saliva, but its effectiveness in xerostomia treatment is still controversial (6-9). While the lubrication of the oral mucosa decreases symptoms, the effects last only for a short duration. Artificial saliva preparations are designed to mimic natural saliva both chemically and physically (10). Electrolytes are added to mimic those in natural saliva and have buffering property, while including calcium, phosphate, and fluoride may provide a remineralizing potential (11).

Currently, the most available artificial saliva formulation mainly utilizes sodium carboxymethylcellulose (SCMC) as an agent with thickening, lubricating, film-forming and mucoadhesive characteristics (12). SCMC which is rapidly hydrated and swollen may not be well retained at the mucosal surface (13). Alternatively, sodium polyacrylate (SPA), a water-soluble polymer with excellent moisture absorption, mucoadhesive and retention ability, is widely used in pharmaceutical formulations and dental applications, primarily to increase viscosity and stabilizing emulsions (14). *Pi*locarpine is a cholinergic parasympathomimetic agonist that binds to the muscarinic-M3 receptors and can cause pharmacological smooth muscle contraction and stimulation of various exocrine glands (15). Topical pilocarpine may be considered as an alternative to minimize the side effects of systemic administration. Previous studies have been performed on the effectiveness of topical pilocarpine as a mouthwash, Tablets, lozenge, spray (16-20), however, the study of topical applications of pilocarpine combined with artificial saliva has not been reported. It is hypothesized that combining topical pilocarpine as an active ingredient with artificial saliva should alleviate xerostomia. Thus, the present study was designed to evaluate the efficacy and safety of two artificial saliva formulations with 0.1% pilocarpine, combined with either, SCMC (SCMC-formula), or, SPA (SPA-formula), for use with xerostomia patients.

## Material and Methods

-Preparation of artificial saliva

Artificial saliva was prepared, composed of potassium chloride 0.07% (Ajax Finechem, Australia), magnesium chloride 0.006% (RCI Labscan, Thailand), calcium chloride 0.02% (Ajax Finechem, Australia), di-potassium hydrophosphate 0.008% (Ajax Finechem, Australia), potassium di-hydrophosphate 0.04% (RCI Labscan, Thailand), and sodium fluoride 0.11% (Ajax Finechem, Australia) as electrolytes and minerals. Xylitol and spearmint oil were used as flavoring agents, and sodium benzoate (Ajax Finechem, Australia) was used as a preservative. Two formulae of artificial saliva were successfully fabricated with mucoadhesive base polymers using either 2.5% of SCMC (Sigma-Aldrich, USA) or 0.25% of SPA (Aronvis SX, Toagosei, Japan). Then, the solutions were sterilized using an autoclave (Daihan, Korea) at 121°C pressure 1 bar for 15 min. After sterilization was complete, pilocarpine hydrochloride concentration 0.1% (Sigma-Aldrich, USA) and spearmint oil were added as saliva stimulating agents. The finished products were kept refrigerated (4-8°C) until use.

-Physico-chemical properties of the developed artificial saliva formulation 

Normal saliva was collected from 3 healthy volunteers (24–25 years old). The volunteers gargled their mouths with drinking water prior to saliva collection. Viscosity, pH and surface tension of the two formulae of artificial saliva and normal saliva are shown in Table 1. The viscosity of the developed artificial saliva formulations was determined by using Brookfield DV II Pro viscometer (Brookfield Engineering Laboratories, Inc., Middleboro MA, USA). Samples were obtained at a rotational speed setting of 20 rpm at room temperature. The surface tension of the artificial saliva was performed by Du Noüy ring method (Du Nouy tensiometer®, USA). The interface of saliva was placed by platinum ring and the maximum force was measure interfacial tension (mM/m). Possible heavy metal contamination and microbial contents were examined in accordance with international standards (U.S. Food and Drug Administration (FDA), 2001; Therapeutic Goods Order No.77, 2008). All samples were sent for contamination testing to the Central Laboratory (Khon Kaen, Thailand), Co, Ltd. The heavy metal analysis was performed by atomic absorption spectroscopy (ICP-AES, optima 4300DV, PerkinElmer Inc.).

**Table 1:**
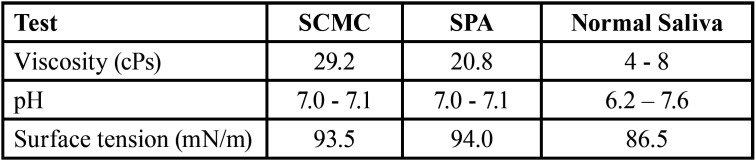
Viscosity, pH and surface tension of two formulae of artificial saliva and normal saliva.

-Testing in healthy volunteers

The two artificial saliva formulae containing either SCMC or SPA, were tested for satisfaction and safety in 40 healthy volunteers. Most of the volunteers gave ratings of satisfied, to, very satisfied, and, none reported adverse events (AEs).

-Study population and methodology 

**Figure 1:**
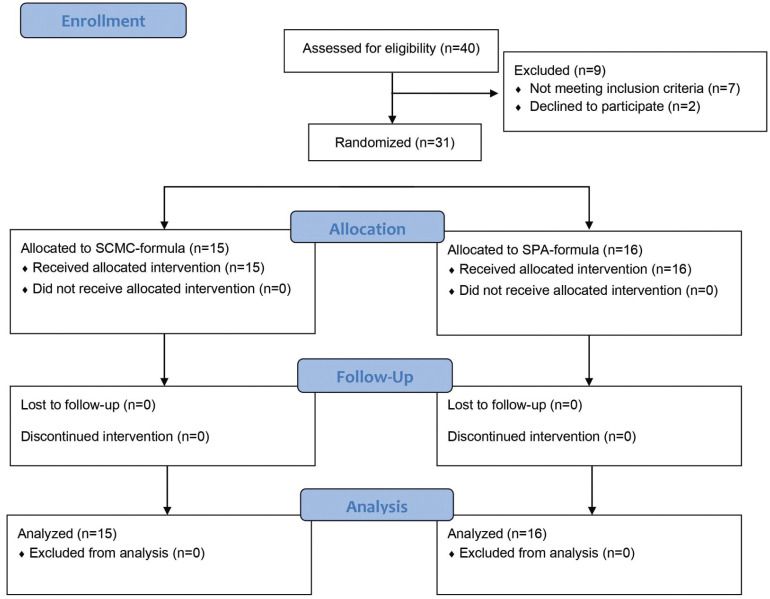
Flow diagram of the study (enrolment, allocation, follow-up, and analysis) performed according to Consolidated Standards of Reporting Trials (CONSORT).

This study used a randomized, parallel, double-blind controlled trial design. Treatment was conducted for 6 weeks to compare the efficacy and safety of the two artificial saliva formulations. Simple randomization, based on a computer-generated random sequence, was used to allocate 31 patients to two groups, group A, (15 patients) (SCMC-formula), or, B, (16 patients) (SPA-formula). Subsequently, both the examiner and the patients were blinded to the type of artificial saliva being used. Participants were enrolled from September 2020 to February 2021, as shown in the study flow diagram (Fig. 1).

-Xerostomia participants

Thirty-one xerostomia patients were recruited from the Oral Medicine Clinic, Faculty of Dentistry, Khon Kaen University, Thailand. The xerostomia could be secondary to any disorder or related to any cause, including, but not limited to, radiochemotherapy and systemic diseases such as diabetes mellitus, scleroderma, systemic lupus erythematosus, and Sjögren’s syndrome, and adverse effects of certain medications. Inclusion criteria were having a Xerostomia Inventory (XI) (21) score of more than 11 (on their experience and level of dry mouth symptoms) and informed consent. Exclusion criteria were pathologic oral lesion, alcohol and caffeine consumption, smoking, sodium benzoate allergy and pilocarpine allergy or having any contraindication. All the patients were informed by the clinicians about their condition and the study and then signed informed consent. Participants were instructed to take 0.5 ml of the artificial saliva, hold it in the mouth for a few minutes, then spit out NPO for 30 minutes, 4 times daily for 6 weeks.

-Data collection

The primary outcome measure was the XI questionnaire for subjective dry mouth scores, while the Clinical Oral Dryness Score (CODs) (22) was used to evaluate objective dry mouth signs, and sialometry was used go to measure whole salivary flow rate. All parameters were evaluated at baseline and after the 6-week treatment, as well as an NRS oral moistness score at the 6th week after treatment with these preparations. After the participant refrained from eating or drinking for 1 hour, unstimulated and stimulated (with 4x4 cm paraffin sheet) whole saliva was collected for 5 min using an established spitting technique. SGH was considered when the salivary flow rate was <0.1 mL/min at rest (1). A secondary outcome assessment for safety was carried out from the start of the study until 6 weeks after administration of the study product. AEs were checked by measuring the participants’ blood pressure and heart rate, including oral examination, self-reported AEs via a questionnaire.

-Data Analysis

Initially, each variable was submitted to the Shapira-Wilk normality test, confirming a non-normal distribution. Therefore, differences between the two groups in the 6th week were analyzed by Mann-Whitney U test. A Wilcoxon Signed Rank Test was used for comparisons between before and after 6-week treatment in both groups. The level of statistical significance was 0.05 (*P*<0.05). Descriptive data were presented as mean±SD values, median and interquartile range (IQR). All statistical analyses were conducted using IBM SPSS statistic software, version 19.

-Ethical Review

The study was performed according to the Declaration of Helsinki and ICH-GCP principles and was reviewed and approved by the Ethics Committee for Human Research of Khon Kaen University (#HE621037). All participants provided written informed consent. The study followed the Consolidated Standards of Reporting Trials (CONSORT) reporting guideline (Fig. 1) and was registered in the ClinicalTrials.gov registry (TCTR20200902001).

## Results

The majority of the 31 participants were females (87.1%), with ages ranging from 39 to 83 years (mean 62.10±10.49). All had xerostomia, and 14 (45.2%) were considered SGH. Hyposalivation was found in 46.7% of the SCMC-treated group, and 43.8% of the SPA-treated group. Symptom duration of xerostomia ranged from 0.25 month to 20 years (mean 33.56±52.99 months). Participants reported 51.6% oral burning sensation, 32.3% dysgeusia, 12.9% xeropthalmia, and 6.5% oral numbness. The most frequently reported co-morbidities were 35.5% hypertension and 32.3% diabetes mellitus, followed by 25.8% hyperlipidemia, 22.6% anxiety/depression, 12.9% history of radiochemotherapy, 6.5% Sjögren’s syndrome, 3.2% scleroderma and 3.2% lupus erythematosus, while 5 participants (16.1%) were otherwise healthy. Participants’ demographic characteristics are shown in Table 2.

**Table 2:**
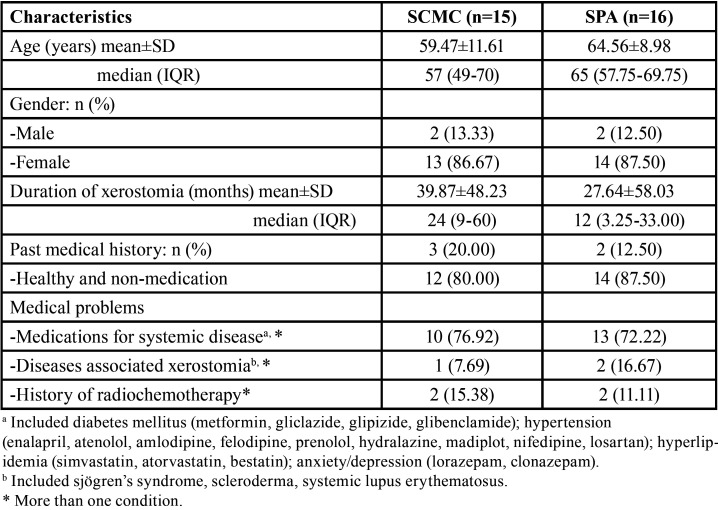
Demographic characteristics of participants.

-Primary outcomes

Both groups post 6-week treatment dry mouth scores and salivary flow rates were compared with their baseline scores. As shown in Table 2, after 6-week treatment, the SCMC-treated group had significantly lower CODs and higher unstimulated and stimulated whole salivary flow rates (*p*<0.001, *p*=0.035, and *p*=0.013, respectively), while the SPA-treated group only showed significantly lower CODs (*p*=0.004). There was no significant difference (*p*>0.05) using XI and salivary flow rates in the SPA-treated group. Additionally, there was no significant difference in dry mouth scores and salivary flow rates between SCMC-treated and SPA-treated groups post the 6 weeks of treatment (*p*>0.05, all) (see Table 3). NRS oral moistness scores for most patients showed 59.3% and 55.3% improvement, respectively, in both SCMC-treated and SPA-treated groups. Five patients (16.1%) had increased salivary flow rates toward the normal saliva flow rates after treatment with these formulations.

**Table 3:**
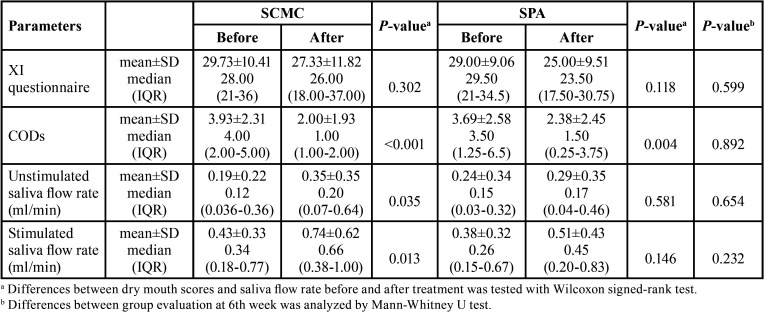
Dry mouth scores and saliva flow rates in participants before and after 6-week treatment using 0.1% pilocarpine in artificial saliva with SCMC or SPA.

-Secondary outcome 

Participants’ blood pressure and heart rate were not significantly affected. However, AEs from artificial saliva with 0.1% pilocarpine formula were observed in 3 participants (9.7%). In the SCMC-treated group, burning tongue was reported in one (6.7%), while one in the SPA-treated group reported dizziness (6.3%) and another had watery eyes (6.3%). No clinically significant abnormalities were detected after using both products, no severe AEs were reported, and no participant withdrew from the study.

## Discussion

In this randomized, parallel, double-blind controlled trial, we found non-significant differences between the SCMC-formula and SPA-formula on subjective dry mouth scores after the 6-week treatment phase, though, both groups reported decreased severity of dry mouth symptoms over this period. However, objective COD measures showed a significant reduction from scores of 4 to 1 for objective dry mouth after treatment for the SCMC-formula, with a similar but non-significant decreasing tendency from score 3.5 to 1.5 for the SPA-treated group. The unstimulated and stimulated whole salivary flow rates after treatment with the SCMC-formula showed significant increases from 0.12 to 0.20, and 0.34 to 0.66 ml/min, respectively. Accordingly, all parameters present showed improved dry mouth scores and salivary flow in both groups, though, with superior efficacy for the SCMC-formula in CODs, on unstimulated and stimulated whole salivary flow rates. It appears both formulations improve xerostomia outcomes. Interestingly, both SCMC-formula and SPA-formula improved hyposalivation toward the normal saliva flow rates (16.1%). Previous studies have commonly used SCMC-based artificial saliva to relieve xerostomia symptoms (6-8) and found SCMC-based saliva substitutes had moderate effects in reducing dry mouth symptoms (8). However, it is difficult to compare across other studies, as different topical forms and parameters of measurement for dry mouth were used.

Previously, a clinical trial using a spray formulation of 1.54% pilocarpine solution for xerostomia (XI) showed no statistical difference in stimulated salivary flow rates between pilocarpine and placebo (20). However, another study demonstrated improvement following the use of topical pilocarpine in moisture sensation and other subjective parameters (9). It has also been reported that xerostomia was relieved by gargling with pilocarpine solution at concentrations of 0.01–2% (16,18,19,23), and by use of a 0.1% pilocarpine mouthwash (18). This concentration of pilocarpine has been reported to produce maximum effect with minimum cost and systemic adverse effects.

SPA, a polymer with superabsorbent property, is transformed into a gel structure and incorporates a large number of water molecules into the meshwork (24). In the SPA-base formulation, we expected better local retention, sustaining the drug on the oral mucosa surface, and improved efficacy regardless of saliva flow. Unfortunately, only one parameter in CODs for the SPA group showed a statistically significant difference after the 6-week treatment. These phenomena have been explained by possible difference in salivary output and underlying salivary gland pathology in the patients studied. In fact, there was no correlation between the degree of increase in the rate of salivary secretion and subjective perception of improvement was found in one study (25). However, in another study, the objective measurement of whole salivary flow rate and subjective xerostomia scores rarely correlated (26). It may be that a slightly lower viscosity of the SPA formula than for SCMC, might actually reduce retention in the oral mucosa. Valid and reliable measurement of objective and subjective dry mouth, including salivary flow rate are also methodological challenges.

Intraoral topical agents are the most common treatment options for the management of xerostomia, to date. Short residence on the mucosal epithelium renders the need to use mucoadhesive ingredients in topical delivery of pilocarpine. Mucoadhesion occurs via electrostatic interactions between SCMC or SPA and glycoproteins of the mucins on the epithelial surfaces. This is the first study that examines formulation combinations with saliva substitutes and salivary stimulants. The SCMC-based and SPA-based artificial saliva are preparations that have composition and physicochemical properties that approximate whole natural saliva, combined with 0.1% pilocarpine, which stimulates salivary gland function and provides longer moisture of the oral mucosa. Improved oral moistness after the 6-week treatment was reported from both SCMC and SPA groups, by 59.3% and 55.3% of the participants, respectively. In fact, there are 2 possible mechanisms to explain these results. First, artificial saliva may act like salivary lubrication, moistening, and buffering. The lubricant effect of artificial saliva may increase oral physiological movement that can promote local mechanical stimuli, along with spearmint oil and xylitol used as flavoring agents which function as salivary stimulants (27). The second, pilocarpine can promote saliva secretion by stimulating muscarinic M3 receptors (15). Pilocarpine is a non-specific stimulation of muscarinic receptors whose, most common side effects are diaphoresis, followed by nausea, palpitation, and tearing (28). This trial presented AEs, possibly resulting from topical pilocarpine being absorbed by oral mucosa then having pharmacologic effects on the exocrine glands, including sweat, salivary, and lacrimal glands. However, there was no serious drug-related AEs evident in the present study.

Limitations of this study, include small sample size and possibility of varying causes of xerostomia across patients. There was large variation of xerostomia in both groups, thus, treatment outcomes may have been related to differing causes of dry mouth and residual salivary gland function. Nevertheless, our pilot study provides evidence of better clinical outcomes for xerostomia from using topical pilocarpine in an artificial saliva preparation. We recommend further studies should more closely examine inclusion criteria for participants, including xerostomia etiology and capacity of salivary gland function, as well as a larger sample size. Further investigation titrating various concentrations of 0.1-2% pilocarpine in artificial saliva preparations might also be fruitful for minimizing adverse effects. However, this is considered as a pilot study, mainly reporting the safety of topical applications of saliva-based polymers containing pilocarpine.

## Conclusions

This randomized controlled pilot trial demonstrated superior efficacy of a SCMC-based artificial saliva preparation containing 0.1% pilocarpine over a SPA-formula over a 6 weeks xerostomia treatment trial. In addition, both formulations were found to be clinically safe with minimal adverse effects.
